# Simultaneous Determination of RDX and HMX in Rat Plasma by LC-MS/MS and its Applications

**DOI:** 10.3389/fchem.2022.808226

**Published:** 2022-02-10

**Authors:** Xi Zhang, Zhuoling An, Yali Lv, Guangrun Li, Lihong Liu, Pengfei Li

**Affiliations:** ^1^ Department of Pharmacy, Beijing Chao–Yang Hospital, Capital Medical University, Beijing, China; ^2^ Department of Pharmacy, China-Japan Friendship Hospital, Beijing, China

**Keywords:** RDX, HMX, LC-MS/MS, plasma, quantitative analysis

## Abstract

**Background:** 1,3,5-trinitroperhydro-1,3,5-triazine (RDX) and octahydro-1,3,5,7-tetranitro-1,3,5,7-tetrazocine (HMX) can cause serious toxicity problems in humans and animals, but direct analyses of RDX and HMX in biological samples are very limited. A rapid and efficient liquid chromatography-electrospray quadrupole linear ion trap mass spectrometry (LC-MS/MS) method suitable for the simultaneous determination of RDX and HMX in rat plasma after intravenous administration of two nitramine compound mixed solutions has been developed.

**Methods:** Plasma samples were pretreated with one-step protein precipitation, the plasma consumption is as low as 100 μl. RDX, HMX, and internal standard mycophenolic acid were eluted for 8.0 min on a reversed-phase C_18_ analytical column with a water/acetonitrile mixture as the mobile phase. An electrospray ionization (ESI) source was applied and operated in negative ion mode. The optimized mass transition ion pairs (m/z) monitored for RDX, HMX, and internal standard mycophenolic acid were *m/z* 284.1→61.7, *m/z* 331.0→108.8, *and m/z* 319.2→191.1, respectively.

**Results:** The detection ranges of both RDX and HMX in plasma were 5.00–200.00 ng⋅ml^−1^ with an LOD of 1.00 ng⋅ml^−1^. The extraction recoveries of RDX and HMX were 60.04 ± 4.18% and 79.57 ± 3.35%, respectively. The precision and accuracy met the requirements, and the method was stable under all tested conditions.

**Conclusion:** The present method is miniaturized, effective, portable, rapid and can be easily used for simultaneous quantification of RDX and HMX in rat plasma.

## Introduction

**FIGURE 1:**
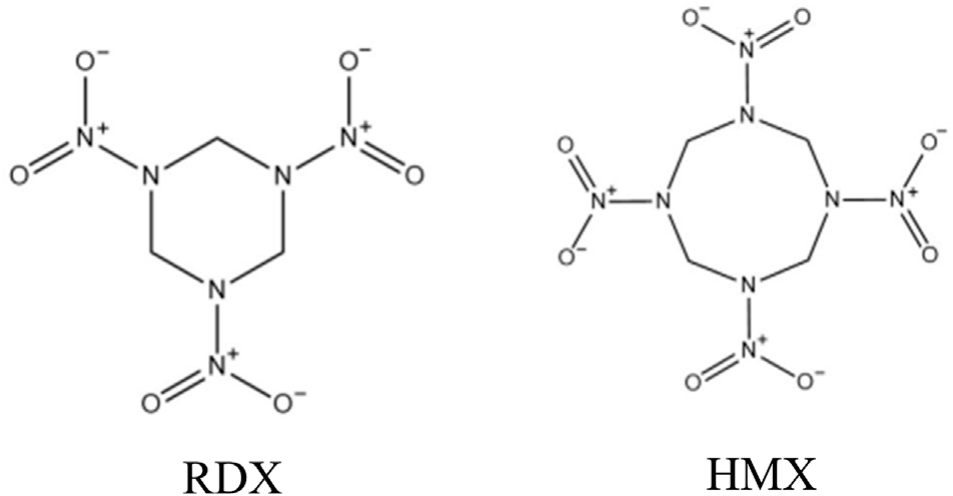
The chemical structures of RDX and HMX.

1,3,5-trinitroperhydro-1,3,5-triazine (RDX) and octahydro-1,3,5,7-tetranitro-1,3,5,7-tetrazocine (HMX) are highly energetic chemicals that have been used for military, industrial, mining, and terrorist activities (1). The chemical structures of RDX and HMX are shown in Figure 1. Their wide usages often lead to the uncontrolled discharge of RDX and HMX into the environment. RDX and HMX are moderately to slightly soluble in water, and thus can migrate through groundwater (Bansal et al., 2012) to cause animals (Bannon et al., 2009; Gust et al., 2009; Williams et al., 2012; Kuperman et al., 2013), plants (Vila et al., 2007) and humans (Johnson et al., 2007) contamination. In addition, they can also adversely affect mammals through inhalation or oral and dermal exposure (Talmage et al., 1999). A series of studies have demonstrated that RDX and HMX can cause central nervous system (CNS), renal, and gastrointestinal (GI) toxicity in humans and animals (Bruchim et al., 2005; Johnson et al., 2007; Quinn et al., 2009; Deng et al., 2014; Garcia et al., 2019; Chong et al., 2020). RDX can even induce aberrant expression of MicroRNAs in mouse brain and liver (Zhang and Pan, 2009). The US Environmental Protection Agency (EPA) classifies RDX as a potential human carcinogen (Class C) (Özhan et al., 2003). Environmental contamination by RDX and HMX have raised health concerns about human and environmental exposure (Zhang and Pan, 2009).

As the pharmacokinetic data of RDX in a three-year-old child from previous study (Woody et al., 1986) showed that it was most discharged from feces in original form, and a small part was discharged from urine in original form. The degree of toxicity was positively correlated with the concentration in blood, which was consistent with other literature results (Özhan et al., 2003; Garcia et al., 2019). Therefore, quantitative detection original form of RDX and HMX *in vivo* is a good method for the treatment and early warning of poisoning. But the currently accepted analytical methods for the determination of nitramines in biological fluids are limited (Woody et al., 1986; Özhan et al., 2003; Bansal et al., 2012). Liquid chromatography combined with UV detection was chosen for these studies and the limited detections were circumscribed. MEPS (Bansal et al., 2012), SPE (Özhan et al., 2003), simple filtration (Woody et al., 1986) were chosen to extract RDX in the literatures. Large amounts of solvent were required for some of these techniques and multi–step procedures led to a loss of analyte. It is important to concentrate on rapid, sensitive,less laborious and economical techniques.

As RDX and HMX can be found in environment, many analytical methods for the determination of trace levels of nitramines in water and soil matrices have been developed, such as micellar electrokinetic chromatography (MEKC) (Brensinger et al., 2016), gas chromatography-mass spectrometry with either electron-capture detection (GC-ECD) (Walsh, 2001), mass spectrometry (GC-MS) (Yinon, 1996), high-performance liquid chromatography (HPLC) equipped with a diode array tube detector (HPLC-DAD) (Borch and Gerlach, 2004), HPLC combined with UV detection (Song and Bartmess, 2009), liquid chromatography-mass spectrometry (LC-MS) (Holmgren et al., 2005; Tachon et al., 2007), and LC-MS/MS (Cassada et al., 1999; Pan et al., 2006). Among the above methods, LC-MS/MS has higher sensitivity and selectivity, whose method detection limit (MDL) of RDX is as low as 0.004 ng⋅mL^−1^ (Cassada et al., 1999). LC-MS/MS is an attractive alternative for explosive analysis.

In this study, a very fast and simple LC-MS/MS method is developed for the analysis of RDX and HMX in rat plasma. The present work is also the first report of the method development for the determination of HMX in biological samples. Compared with previous detectors, the present method with ESI–MS/MS has distinct advantages. An easy sample pretreatment leads to less laborious, less time-consuming and low amounts of organic solvent. The whole method was applied to a model sample collected from contaminated laboratory rats. Here, rat is just used as an example, the method should may also be applied in humans or other animals.

## Materials and Methods

### Materials and Reagents

RDX (1.00 mg⋅ml^−1^, 98.6%) and HMX (1.00 mg⋅ml^−1^, 98.1%) were purchased from AccuStandard, Inc. (Beijing, PR China). Mycophenolic acid was purchased from Sigma-Aldrich (Beijing, PR China). Acetonitrile was HPLC grade and purchased from Fisher Scientific (Fair Lawn, NJ, United States). All other chemicals were of analytical grade and used without further purification. Distilled demineralized water was produced by a Milli-Q Reagent Water System (Millipore, MA, United States).

### Animals and Sample Collection

Eight male Sprague-Dawley rats (approximately 180–200 g) were obtained from the Department of Animal Science, Peking University Health Science Center. Prior to the initiation of the study, all rats were raised at room temperature (25 ± 2)°C and relative humidity (50 ± 10) % under a 12 h light/dark cycle and had free access to tap water and pelleted rodent chow for 3 days. Then the rats were intravenously injected with 0.5 ml nitramine compound mixed solution, which had been prepared by dissolving nitramine compounds in water. The concentrations of RDX and HMX were 100.00 μg⋅ml^−1^ and 200.00 μg⋅ml^−1^, respectively. Blood was drawn into 1.5 ml Eppendorf tubes containing heparin sodium from ophthalmic veins of each rat before (0 h) dosing and at 5, 15, 30 min, 1, 2, 4, 6, and 8 h after that. The plasma samples of rats were collected from blood by centrifugation at 4,000 rpm for 10 min and stored at 20°C prior to analysis.

### Sample Preparation

The stock solution of internal standard (IS) (200.00 ng⋅ml^−1^) was prepared by dissolving 7.14 mg mycophenolate reference substance (equivalent to 7.00 mg pure mycophenolate) in a 7 ml volumetric flask and diluting to 200.00 ng⋅ml^−1^ working solutions with acetonitrile. An aliquot of 100 μl plasma sample was transferred into a 1.5 ml Eppendorf tube, 200 μl of IS working solution and 100 μl acetonitrile were added to remove proteins, and the mixture was vortex for 1 min and centrifuged at 13,200 rpm for 10 min. The supernatant was transferred into a 200 μl autosampler vial and injected into the instrument for analysis by LC-MS/MS with a 5 μl injection loop.

### Instrumentation

The LC-MS/MS system consisted of an Agilent 1,100 series HPLC (Agilent Technologies, Palo Alto, CA, United States) coupled to an Applied Biosystems Sciex 3200Qtrap™ mass spectrometer (Applied Biosystems Sciex, Ontario, Canada). Applied Biosystems/MDS SCIEX Analyst software was applied for data acquisition and processing.

### Chromatographic Conditions

Gradient elution chromatography (as shown in Table 1) was carried out at 25°C on a 150 mm × 4.6 mm, 5 μm Zorbax Eclipse HC-C_18_ column (Agilent Technologies, Palo Alto, CA, United States) maintained using the mobile phase of acetonitrile and water at a flow rate of 1.0 ml⋅min^−1^. The column effluent was split so that approximately 0.5 ml⋅min^−1^ entered the mass spectrometer. The LC analysis total runtime was 8 min and the retention time was 3.69 min for RDX, 3.62 min for HMX, and 3.81 min for mycophenolic acid.

**TABLE 1:** The conditions of gradient elution.

Time (min)	Flow rate (ml⋅min^−1^)	Acetonitrile (%)	Water (%)
0.00	1.00	15.00	85.00
0.20	1.00	15.00	85.00
1.20	1.00	95.00	5.00
4.50	1.00	100.00	0.00
4.51	1.00	15.00	85.00
8.00	1.00	15.00	85.00

### Mass Spectrometer Conditions

An electrospray ionization (ESI) source was used in negative ion mode for all experiments. The LC-MS/MS detector was operated at low resolution in MRM mode using the mass transition ion pair *m/z* 284.1→*m/z* 61.7 for RDX, *m/z* 331.0→*m/z* 108.8 for HMX, and *m/z* 319.2→*m/z* 191.1 for mycophenolic acid. MS parameters were optimized as follows: curtain gas, 20 units; gas 1, 50 units; gas 2 (nitrogen), 60 units; dwell time 200 ms; source temperature 580°C; ion spray voltage −4,500 V. Declustering potential (DP) and collision energy (CE) were −16 V and −26 eV for RDX, −25 V and −19 eV for HMX, and −35 V and −32 eV for mycophenolic acid, respectively. The collision gas was set to medium mode, and the interface heater was set to “on” mode.

Hydrophilic impurities were diverted to waste for 2.1 min after an injection using a ten-way switching valve. Data acquisition was carried out by Analysis 1.5.1 software on a *Dell* computer.

### Assay Validation

The mixed stock solution of RDX and HMX was freshly prepared by spiking RDX (1.00 mg⋅ml^−1^) and HMX (1 mg⋅ml^−1^) into acetonitrile. The solution calibration curves consisted of seven concentration levels, which were prepared by spiking the appropriate mixed solution of nitramines into blank plasma at concentrations of 5.00, 10.00, 20.00, 50.00, 100.00, 150.00 and 200.00 ng⋅ml^−1^. Low-, medium- and high-QC samples of plasma (20.00, 100.00, 160.00 ng⋅ml^−1^) were prepared by spiking the appropriate solution standards into blank plasma. It must be noted that all mixed solutions and QC samples should be kept away from direct sunlight. In each analytical run, calibration standards, QC samples, and plasma samples were extracted together.

Calibration standards and QC samples for plasma (n = 6) were analyzed on three separate days. The linearity of the calibration curves based on peak areas were assessed by weighted (1/x^2^) least-squares nonlinear integral analysis (y = ax^2^+bx + c as regression model). The limit of detections (LODs) for RDX and HMX in the plasma matrix were determined as the signal corresponding to three times the background noise on each mass chromatogram. The limit of quantifications (LOQs) for RDX and HMX in the plasma matrix were determined as the signal corresponding to ten times the background noise on each mass chromatogram. The precision and accuracy of the method were evaluated in terms of intra-day and inter-day precision. The coefficient of variation (CV) was calculated as repeatability, and the relative error (RE) was calculated as accuracy. The reproducibility was calculated in the same way but by repeating the procedure over a range of 3 days. The absolute recovery rates of RDX and HMX were evaluated by the ratio of the calculated concentration of blank plasma spiked with QC samples to their nominal concentration. The test of absolute recovery rates contained three samples at each concentration.

The stabilities of RDX and HMX in plasma were assessed both at −20°C for 60 days and after three freeze-thaw cycles. After extraction and reconstitution, the stabilities of the QC samples in plasma were also tested in the autosampler at room temperature for 0, 2, 4 h, and two successive times. The samples for stability tests were quantified using freshly prepared calibration standards.

### Application

The proposed LC-MS/MS analytical method was used in eight rat plasma samples. The concentrations were quantified over a period of 0–8 h, and the average concentration-time curves of RDX and HMX in the treatment group were drawn.

## Results and Discussion

### Mass Spectrometry

**FIGURE 2:**
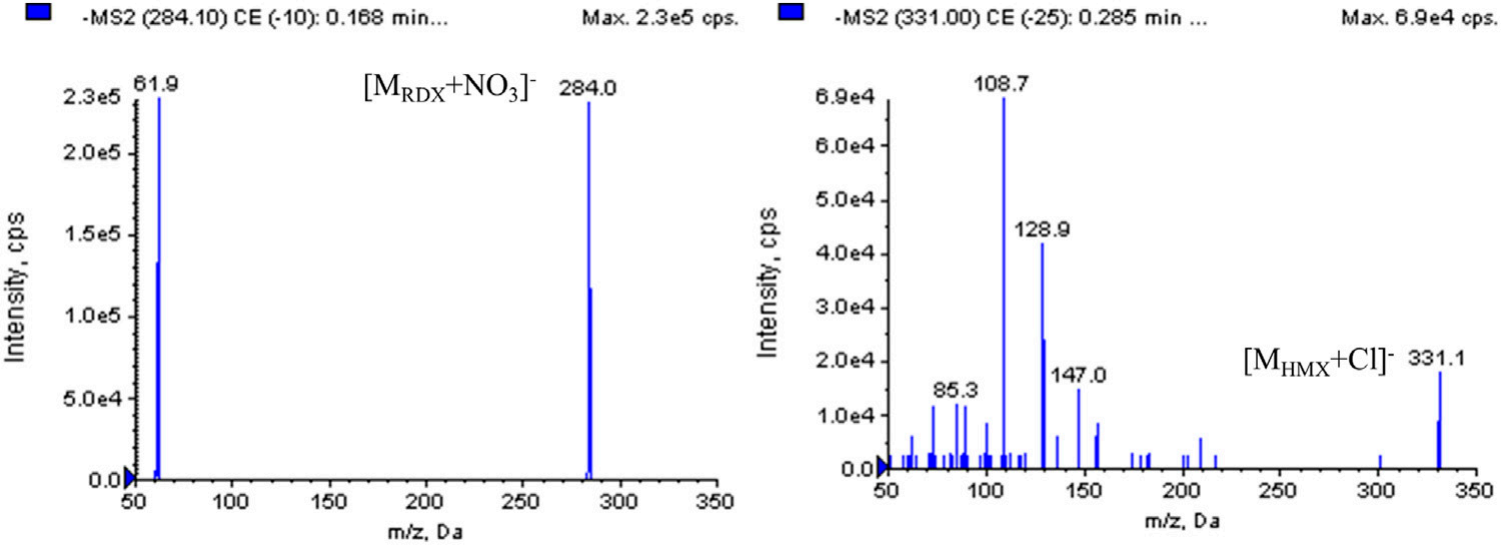
The second-fragment mass spectra of RDX, HMX and IS.

Our preliminary findings during the method development showed that the electrospray source (ESI) was determined to be more sensitive to nitramines than APCI, and ESI was chosen for the ionization of the analytes. Instrumental tests with standard solutions in positive ion detection mode produced little response, presumably because of the low proton affinity of the nitramine compounds. ESI in negative mode is more suitable for nitramine analysis by LC-MS. According to the special chemical structures of nitramines, full-scan negative mode spectra of RDX and HMX contained an array of complicated adduct ions that were formed from their own fragment ions or impurities in the formation. In this study, additive solutions such as ammonium acetate, formic acid, and ammonium hydroxide were employed and tested to achieve unequivocal recognition of RDX and HMX. However, the expected adduct ions were not dominant and had a lower response. Without any additives, the ESI mass spectra of RDX and HMX produced an intense ion forming the base peak at *m/z* 284.1 [M+62]^-^ for RDX and at *m/z* 331.0 [M+35]^-^ for HMX. These ions were interpreted as [M_RDX_ + NO_3_]^-^ and [M_HMX_ + Cl]^-^, which were well characterized and reproducible, presumably generated from impurities in the extraction solution or formation. Adduct ion spectra of [M_RDX_ + NO_3_]^-^ and [M_HMX_ + Cl]^-^ showed the fragment ions of RDX at *m/z* 61.9 and that of HMX at *m/z* 108.8 (Figure 2), which were present in the highest abundance and were chosen for the multiple reaction monitoring (MRM) acquisition of RDX and HMX.

### Chromatography Spectrometry

Acetonitrile/water was finally chosen because its greater chromatographic selectivity enabled better separation and higher response of nitramines. Other parameters, especially the flow rate and column temperature, were optimized. By optimizing the flow rate of 1.0 ml⋅min^−1^, the cycle time of a single sample was reduced to 8 min which is 2 minutes shorter than that of the previous study (Özhan et al., 2003) and the same as that of Pooja Bansal’s study (Bansal et al., 2012), allowing a throughput of 100–120 samples per day. Among the different column temperatures tested (25, 45, 60°C), the best chromatographic signal-to-noise (S/N) ratio was obtained at 25°C. Under those conditions, the retention time without any co-elution interferences was typically 3.69 min for RDX, 3.62 min for HMX, and 3.81 min for mycophenolic acid.

### Sample Preparation

The pre-treatment methods in previous studies included MEPS (Bansal et al., 2012) and SPE (Özhan et al., 2003). Although both methods achieved good extraction recovery, the MEPS was expensive, time-consuming, tedious and difficult to operate because 100 μl plasma was drawn slowly (approximate rate 20 ± 5 μl s^−1^) through the syringe for ten times and then the BIN enrichment process need 5–8 min (Bansal et al., 2012). Large amounts of solvents were required for SPE which were expensive, health hazardous and harmful to environment, at the same time, the extraction time of each sample was 20 min (Özhan et al., 2003). In this study, the one-step protein precipitation was finally chosen because it is rapid and simple handled. Methanol and acetonitrile were tried as protein precipitation solvents during the method development, acetonitrile was finally chosen because it ensures a more extended recovery yield. With the One-step protein precipitation, the volume of the reagent-consuming was as low as 300 μl and the whole process took only 10 min.

### Selection of IS

To obtain better accuracy and precision, both telmisartan and mycophenolic acid were tested as IS. Mycophenolic acid was finally chosen because it reduced both the matrix effects on analytes, and the effect of volume variability during sample extraction more efficiently, although not completely.

### Assay Validation

#### Selectivity

**FIGURE 3:**
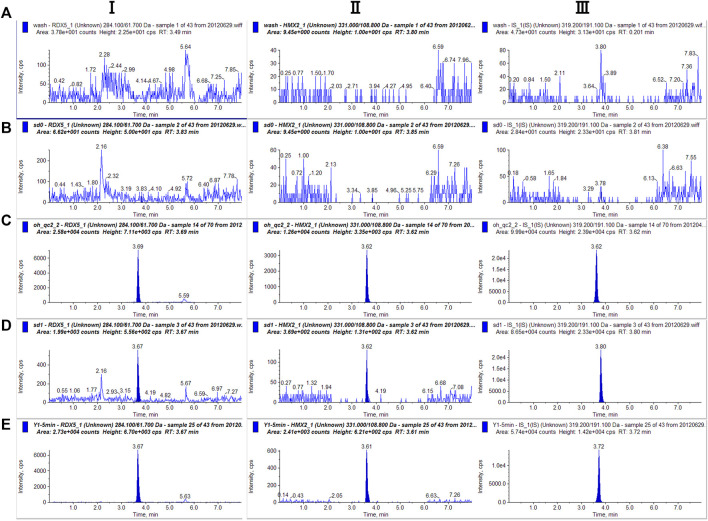
Representative single reaction monitoring chromatograms of **(A)** blank solution **(B)** blank plasma **(C)** blank solution spiked with RDX (100.00 ng⋅ml^−1^), HMX (100 ng⋅ml^−1^) and IS **(D)** blank plasma spiked with RDX (100 ng⋅ml^−1^), HMX (100.00 ng⋅ml^−1^) and IS (E) a plasma sample 0–5 min after intravenous 0.5 ml mixted nitramines compounds (RDX 100.00 μg⋅ml^−1^, HMX 200.00 μg⋅ml^−1^).

The chromatograms of blank solution, blank solution spiked with samples, blank plasma, and the corresponding standard plasma spiked with samples are shown in Figure 3. By observing and comparing those chromatograms, no endogenous interferences were found during the retention time of either analyte or IS. Therefore, there was no plasma background interfering with the measurement of RDX, HMX, and IS, demonstrating the selectivity and specificity of the MRM technique.

### Linearity and Dynamic Range

Our preliminary findings during the method development showed that serious ion suppression of the electrospray signal of analytes existed at high concentrations regardless of the amount of applied ion voltage. This led to the poor linearity of the calibration plots in the plasma matrix, presumably because the analytes were more easily solvated than the interferences in the plasma matrix, and their speed of evaporation from the ESI droplet was seriously suppressed by the interferences. The instrumental calibration plots (calibration plots in solvent) were adapted as calibration standards. The calibration plots were constructed by applying the weighted (1/x^2^) least-squares nonlinear integral method (y = ax^2^+bx + c as regression model). As Table 2 shows, the linearity of the dynamic range was verified between 5.00 and 200.00 ng⋅ml^−1^ for both RDX and HMX. The calibration curves, containing RDX and HMX standards, were prepared at seven concentration levels (200.00, 150.00, 100.00, 50.00, 20.00, 10.00, 5.00 ng⋅ml^−1^). The typical linear regression equations of the calibration curves were as follows:

$$\text{RDX}:y=5\text{.}62e^{\mathrm{-0.06}}x^{2}+\text{0}\text{.}00439x+\text{0}\text{.}00545\left(r^{2}=\text{0}\text{.}9966\right)$$

$$\text{HMX}:y=1\text{.}03e^{\mathrm{-0.06}}x^{2}+\text{0}\text{.}00151\text{x}+\text{0}\text{.}0013\left(r^{2}=\text{0}\text{.}9936\right)$$

The *y* represents the ratio of the peak area of the analytes to that of the IS, while the *x* represents the concentration of the analytes. The regression coefficients for all calibration plots were no less than 0.9900, demonstrating good linearity in the stated concentration ranges.

**TABLE 2:** LODs, LOQs and line ranges for determination of RDX and HMX in rat plasma.

Analyte	LOD (ng⋅ml^−1^)	LOQ (ng⋅ml^−1^)	Line range(ng⋅ml^−1^)
RDX	1.00	5.00	5.00–200.00
HMX	1.00	5.00	5.00–200.00

The LODs for RDX and HMX in the plasma matrix were determined at the concentration when the signal-to-noise ratio reached three. The LODs values were 1.00 ng⋅ml^−1^ for both RDX and HMX.

As the previous studies reported, the quantitative detection ranges of RDX were 1.00–500.00 ng⋅ml^−1^ (Bansal et al., 2012) and 10.00–2000.00 ng⋅ml^−1^ (Özhan et al., 2003). Compared with previous studies, the quantitative detection scope of this study is narrow, but the LOD is the lowest. Maybe the dilutive test is a effect way to expand the detection range to match the applicable for high concentration samples exceed the upper limit of detection.

#### Method Precision and Accuracy

As Table 3 shows, the intra- and inter-day variability by relative standard deviation (RSD) was 8.16–9.95% and 5.10–8.13% for RDX and 6.14–9.70% and 5.01–12.72% for HMX, respectively. The accuracy ranged from 81.80 to 115.63% for RDX and 83.30–115.00% for HMX. The above values were within the ±20% range, manifesting good precision and accuracy.

**TABLE 3:** Precision and accuracy for determination of RDX and HMX in rat plasma.

Analyte	Nominal conc. (ng⋅ml^−1^)	Calculated conc. (ng⋅ml^−1^)	Intra-day RSD (%)	Inter-day RSD (%)	RE (%)
RDX	20.00	20.01	8.16	5.94	0.06
	100.00	101.12	9.95	8.13	1.12
	160.00	158.89	9.95	5.10	-0.69
HMX	20.00	20.69	6.14	12.72	3.47
	100.00	101.53	9.70	5.01	1.53
	160.00	158.50	8.56	7.09	-0.94

#### Recovery, Matrix Effects, and Stability Studies

A 100% extraction recovery for RDX and HMX was expected because they are polar compounds with no plasma protein binding. To our surprise, the experiments proved that the absolute recovery coefficients were 60.04 ± 4.18% and 79.57 ± 3.35% for RDX and HMX, respectively, in rat plasma. Repeat experiments confirmed this finding. Maybe the majority of loss during extraction is due to association of polar compound with the protein precipitate. This phenomenon was consistent with literature report (Holleran et al., 2020) in which that the extraction recovery for polar compound iohexol was only approximately 50%. Different ratios of acetonitrile to plasma (1 to 5) were tested. We next confirmed that the recoveries were best at 3 ratio of acetonitrile to plasma. As the solution calibration curves consisted of seven concentration levels and low-, medium- and high-QC samples were prepared by spiking the appropriate solution standards into blank plasma. The absolute concentrations of RDX and HMX in rat plasma samples can be obtained by the calculated concentrations divided by the recovery coefficients. The assay using the described protein precipitation with acetonitrile was completely validated and the imperfect recoveries do not affect the accuracy of the method.

The results (Table 4) suggested that both RSD and RE met the requirements and that RDX and HMX in plasma were stable under all conditions.

**TABLE 4:** The stability data of RDX and HMX (n = 3).

Analyte		RDX				HMX			
Storage conditions	Concentration (ng⋅ml^-1^)			RSD (%)	RE (%)	Concentration (ng⋅ml^−1^)		RSD (%)	RE (%)
	Normal		Calculated			Normal	Calculated		
Freezing for 20 days	20.00		18.76	11.27	−6.22	20.00	17.93	9.02	−10.38
	100.00		103.98	1.39	3.98	100.00	104.31	12.33	4.31
	160.00		157.34	5.26	−1.66	160.00	145.55	5.72	-9.03
Three free-thaw cycle	20.00		18.98	4.25	−5.12	20.00	19.32	7.89	−3.42
	100.00		93.69	10.24	−6.31	100.00	93.14	7.38	−6.86
	160.00		160.12	2.54	0.07	160.00	147.69	7.99	−7.69
Stability 0 h	20.00		21.05	9.70	5.23	20.00	19.96	9.70	−0.21
	100.00		102.21	5.10	2.21	100.00	103.05	4.68	3.052
	160.00		148.14	0.41	−7.41	160.00	155.84	4.60	−2.60
Stability 2 h	20.00		19.58	11.85	−2.09	20.00	22.71	13.55	13.54
	100.00		101.63	0.99	1.63	100.00	99.16	2.33	−0.84
	160.00		170.12	2.98	6.32	160.00	162.99	1.89	1.87
Stability 4 h	20.00		21.16	7.40	5.81	20.00	20.17	12.95	0.83
	100.00		100.35	13.65	0.35	100.00	103.67	10.65	3.64
	160.00		159.07	4.93	−0.58	160.00	164.29	4.02	2.68
The first injection	20.00		19.90	2.85	−0.50	20.00	20.95	2.50	4.75
	100.00		108.50	3.54	8.50	100.00	130.00	2.89	9.00
	160.00		164.50	3.09	2.81	160.00	176.50	1.33	10.31
The second injection	20.00		22.70	2.85	13.50	20.00	21.80	7.07	9.00
	100.00		107.50	2.12	7.50	100.00	109.50	3.54	9.50
	160.00		166.50	5.74	4.06	160.00	172.00	3.54	7.50

### Application

**FIGURE 4:**
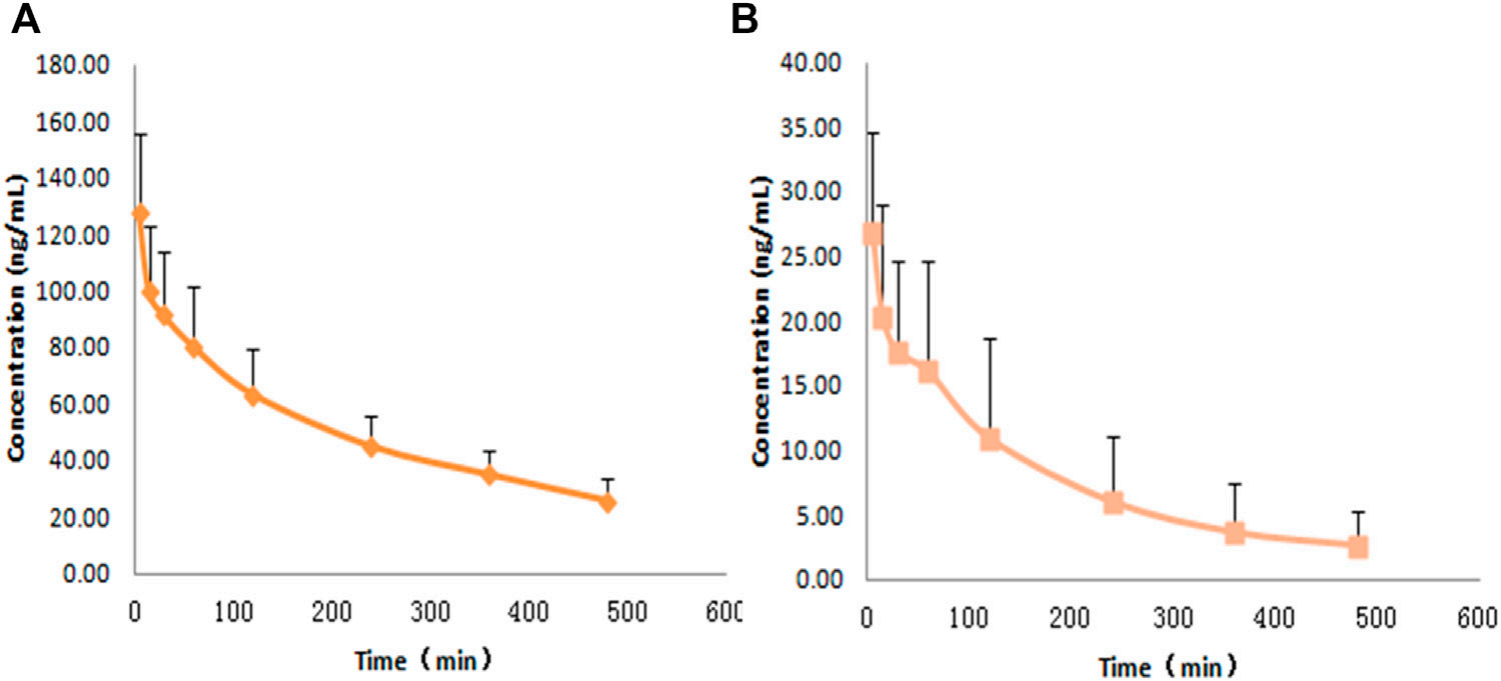
Average plasma concentration-time profiles for RDX and HMX with a single dose of 0.5 ml nitramines compounds intravenously pumping to rats (n = 8) ,**(A)** RDX, **(B)** HMX.

The concentrations of rat plasma samples were quantified by the LC-MS/MS analytical method. The concentrations of RDX were 141.72 ng⋅ml^−1^ at 5 min and 14.69 ng⋅ml^−1^ at 8 h after intravenous administration. The concentrations of HMX were 30.39 ng⋅ml^−1^ at 5 min and 1.24 ng⋅ml^−1^ at 8 h after intravenous injection. The concentration-time curves of RDX and HMX are shown in Figure 4.

## Conclusion

This research takes advantage of the combination of LC-MS/MS, known for its inherent sensitivity and selectivity, and one-step protein precipitation for plasma samples. A quantitative method for the simultaneous determination of RDX and HMX has been developed, optimized, and validated in the first time. For a single sample, the one-step protein precipitation had a consumption of plasma volumes as low as 100 μL and reduced the pretreatment time, proving the pretreatment method is the most advanced up to now. With the ESI-MS detector, the detection ranges of both RDX and HMX in plasma were 5.00–200.00 ng⋅ml^−1^ with LODs of 1.00 ng⋅ml^−1^, so the quantitative method was miniaturized, effective, portable, and rapid. All in all, this LC-MS/MS method is suitable for the trace determination of nitramines in biological fluids. It will be a clinically useful tool for the treatment and early warning of nitramines poisoning.

## Data Availability

The original contributions presented in the study are included in the article/supplementary material, further inquiries can be directed to the corresponding authors.
